# Ferromagnetic behaviour of ZnO: the role of grain boundaries

**DOI:** 10.3762/bjnano.7.185

**Published:** 2016-12-07

**Authors:** Boris Borisovich Straumal, Svetlana G Protasova, Andrei A Mazilkin, Eberhard Goering, Gisela Schütz, Petr B Straumal, Brigitte Baretzky

**Affiliations:** 1 Karlsruher Institut für Technologie, Institut für Nanotechnologie, Hermann-von-Helmholtz-Platz 1, 76344 Eggenstein-Leopoldshafen, Germany; 2 Institute of Solid State Physics, Russian Academy of Sciences, Ac. Ossipyan str. 2, 142432 Chernogolovka, Russia; 3 Max-Planck-Institut für Intelligente Systeme, Heisenbergstrasse 3, 70569 Stuttgart, Germany; 4 National University for Research and Technology “MISiS”, Leninsky prospect 4, 119991 Moscow, Russia; 5 Institute of Metallurgy and Materials Science, Russian Academy of Sciences, Leninsky prospect 49, 117991 Moscow, Russia

**Keywords:** ferromagnetism, grain boundaries, zinc(II) oxide (ZnO)

## Abstract

The possibility to attain ferromagnetic properties in transparent semiconductor oxides such as ZnO is very promising for future spintronic applications. We demonstrate in this review that ferromagnetism is not an intrinsic property of the ZnO crystalline lattice but is that of ZnO/ZnO grain boundaries. If a ZnO polycrystal contains enough grain boundaries, it can transform into the ferromagnetic state even without doping with “magnetic atoms” such as Mn, Co, Fe or Ni. However, such doping facilitates the appearance of ferromagnetism in ZnO. It increases the saturation magnetisation and decreases the critical amount of grain boundaries needed for FM. A drastic increase of the total solubility of dopants in ZnO with decreasing grain size has been also observed. It is explained by the multilayer grain boundary segregation.

## Review

### Introduction

In 2000 the seminal work of Tomasz Dietl et al. appeared [1]. In this work it was predicted theoretically that many semiconductor oxides can become ferromagnetic (FM) if one dopes them with “magnetic” atoms such as iron, manganese and cobalt. Other theoreticians published in that time similar works [2]. It has been predicted that the Curie temperature of such diluted doped magnetic semiconductor oxides can be quite high, even above room temperature. Especially promising was zinc oxide. According to Dietl, ZnO should possess the highest Curie temperature [1].

Of course, such a prediction could not be ignored by experimentalists in the field of semiconductors, because the possibility to make a transparent broadband semiconductor, such as ZnO, ferromagnetic is very promising for future spintronic applications. The ferromagnetism (FM) opens a way to change the optical and/or electrical properties of such a material by applying an external (permanent or alternating) magnetic field. And vice versa, by applying an external (permanent or alternating) electric field one could influence the magnetic behaviour of such a material. Especially attractive is that zinc oxide is cheap. It is widely used for various applications from sunblock creams to varistors for power electronics [3–4]. The various technologies of deposition of pure and doped ZnO films, sintering of ZnO ceramics and growth of single crystals, are well known and well elaborated. It seemed that nothing could prevent the success of the synthesis of ferromagnetic ZnO doped by iron, manganese, cobalt, or other “magnetic” atoms. Indeed first successes came soon. Ferromagnetic ZnO films were synthesised by pulsed laser deposition (PLD), or magnetron sputtering [5–9]. However, the first disappointments also appeared immediately. Namely, single crystals, ceramics sintered from coarse-grained powders and single-crystalline films deposited by molecular beam epitaxy (MBE) were never ferromagnetic. Other synthesis technologies such as wet-chemistry methods or chemical vapour deposition (CVD) sometimes yielded ferromagnetic ZnO and sometimes they did not.

It was of course a challenge for solid-state physics and materials science to explain such strange behaviour and to develop the methods to predict (at least qualitatively) where and when the ferromagnetism appears in zinc oxide. We supposed that ferromagnetic behaviour of pure and doped ZnO is controlled by grain boundaries (GBs) and appears only if the grain boundary network (the “ferromagnetic foam”) is dense enough [7]. Our first results concerning the role of grain boundaries in the ferromagnetic behaviour of pure ZnO and ZnO doped by Mn and Co as well as concerning the dependence of the solubility of Mn and Co in ZnO on the grain size were published in two reviews [10–11] summarizing the essential findings obtained at that time. Later, the hypothesis about the role of GB in ferromagnetic behaviour was supported by our new results on ZnO doped with nickel and iron [6,9,12] as well as by measurements with low-energy muon spin relaxation combined with molecular dynamics modeling and density functional theory calculations [13]. These new results constitute the additional contribution of the current review. It aims to give the comprehensive and updated view on the GB contribution to the ferromagnetic behaviour of ZnO as well as on the multilayer GB adsorption drastically increasing the overall dopant solubility in ZnO. This review is also a modest tribute to the 75th anniversary of Professor Herbert Gleiter who contributed so much to the development of our knowledge of structure, physics and chemistry of grain boundaries.

### Critical grain size for the ferromagnetic behaviour of ZnO

**Figure 1:**
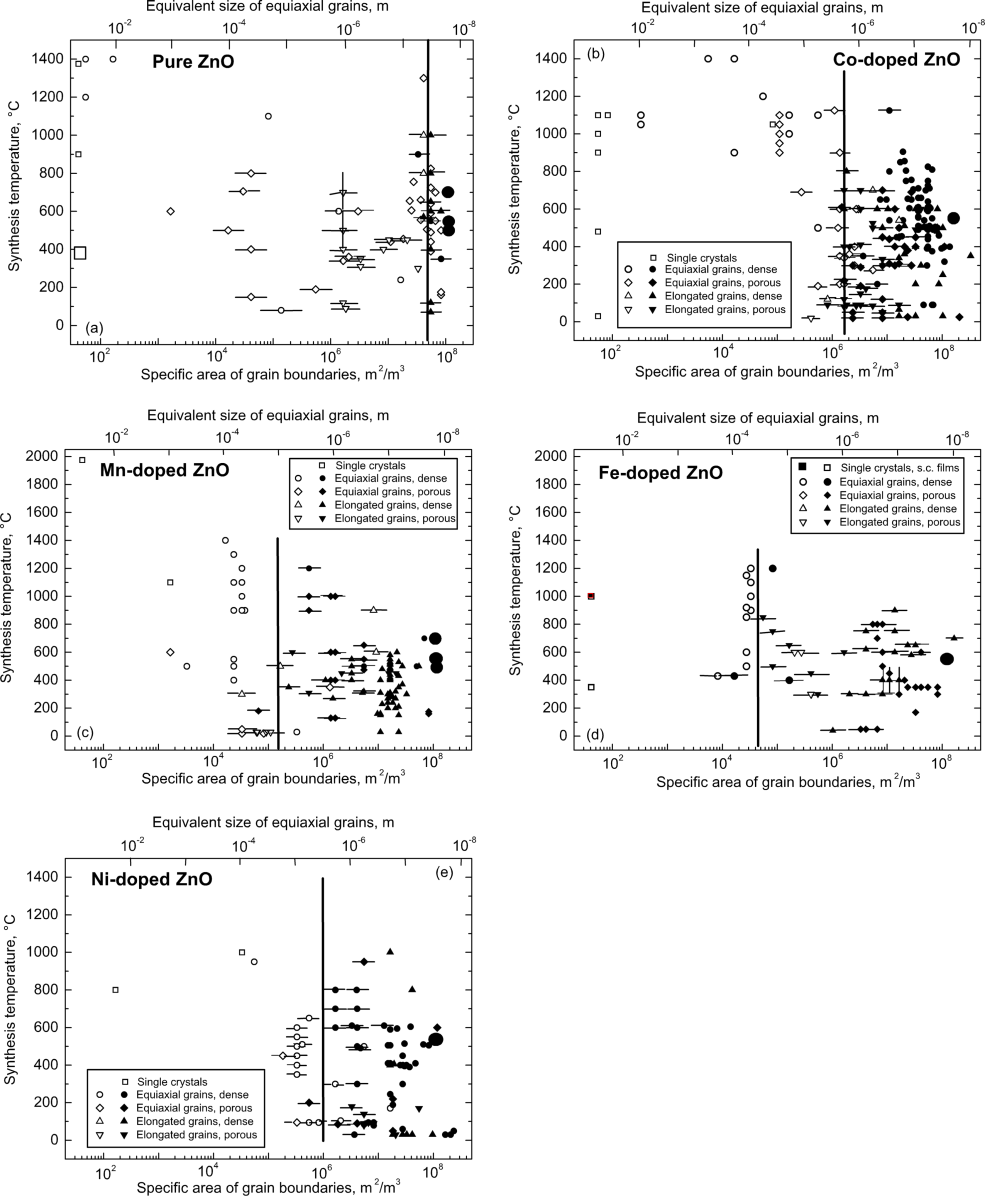
Ferromagnetic (full symbols) and paramagnetic or diamagnetic properties (open symbols) of (a) pure zinc oxide [7] and ZnO doped with (b) cobalt [8], (c) manganese [7], (d) iron [9] and (e) nickel [6] versus the specific area of grain boundaries *s*_GB_ (ratio of the area of the boundaries to the volume) at various synthesis temperatures *T*. In the upper horizontal axis the values of grain size are given as recalculated from *s*_GB_ supposing that the sample is dense and grains are equiaxial. Vertical lines mark the threshold values of *s*_th_ dividing FM (right) and non-FM behaviour of ZnO. Large symbols correspond to the experimental data obtained in the works [6–9]. Figure was replotted basing on the plots reproduced with permission from [6–9], copyright Institute of Problems of Mechanical Engineering, Russian Academy of Sciences (PME RAS, "Advanced Study Center" Co. Ltd), American Physical Society and Taylor & Francis.

First of all we analysed the whole corpus of published data on ferromagnetic behaviour of zinc oxide and developed our own method for the synthesis of pure and doped nanocrystalline ZnO films. The obtained data are summarized in Figure 1 for pure ZnO and ZnO doped with manganese, cobalt, iron and nickel [6–9]. The full list of used references can be found in [6–9]. In each of the five parts of Figure 1 the temperature is plotted along the vertical axis. It is either the synthesis temperature or the temperature of last annealing of the oxides. The grain boundary specific area *s*_GB_ is given in the horizontal axis. *s*_GB_ is the area of GBs in a unit volume. We added an experimental point to the diagrams in Figure 1 if it was possible to estimate from the published experimental work (a) the grain size, (b) the grain shape – equiaxial, elongated or flattened – and (c) porosity of sample, i.e., the portion of grain boundaries and free surfaces. When possible, we tried also to take into account the so-called grain boundary character [14]. In other words we tried to include the high-angle grain boundaries and to exclude the low-angle ones [15]. It is easy to calculate *s*_GB_ if the grains are equiaxial (circles in Figure 1).

The optimal space-filling grain shape for such polycrystals with a minimal surface area is the tetrakaidecahedron, a polyhedron with 14 faces. Thus, the GB-area-to-volume ratio is *s*_GB_ = 1.65/*D*, where *D* is the mean grain size [16]. If the grains were elongated or flattened, the aspect ratio was taken in the account, and *s*_GB_ was modified accordingly (triangles in Figure 1). The equation for *s*_GB_ from [16] is true if a sample is dense and does not contain any pores. In case of porous samples (like for example for partially sintered powders or nanowires, diamonds and downward triangles in Figure 1) the value of *s*_GB_ was multiplied by the porosity factor *p* < 1. In the upper horizontal axis the values of grain size are given as recalculated from *s*_GB_ supposing that the sample is dense and the grains are equiaxial. Squares correspond to the single crystalline samples. They do not contain any GBs, therefore, we put them in the diagram at grain size of 10^−1^ m. Filled symbols in Figure 1 show the data where the ZnO samples were ferromagnetic. Open symbols correspond to the samples for which no FM behaviour has been observed.

**Figure 2:**
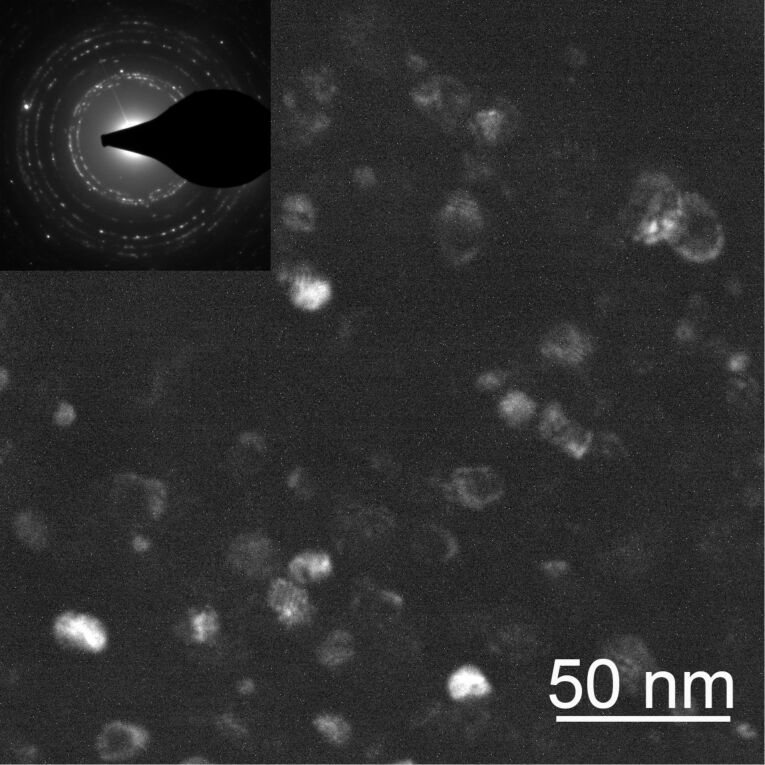
Dark-field TEM micrograph of a thin zinc oxide nanocrystalline film obtained using the liquid-ceramics method.

Large filled circles show our own experimental data obtained using ZnO films synthesized using the original “liquid ceramics” method. This is a kind of so-called wet-chemistry methods for the synthesis of nanograined oxide films. The precursor was zinc(II) butanoate dissolved in an organic solvent. It was used for the preparation of pure zinc oxide. Similar solutions of Mn, Co, Ni and Fe butanoates of were also prepared. The Zn precursor was mixed, respectively, with Mn, Co, Ni or Fe butanoates in appropriate proportions (in order to obtain doped ZnO with dopant contents from 0.1 to 50 atom %). The mixture of liquid precursors was deposited on a substrate (aluminium polycrystalline foil or sapphire single crystalline plate). Then the deposited liquid mixture was dried at 150 °C. After drying the pyrolysis took place in argon or in air at temperatures between 500 and 600 °C. The resulted pure and doped ZnO films of thicknesses between 50 and 200 nm (measured by electron-probe X-ray microanalysis and transmission electron microscopy) were dense (i.e., pore free) and contained equiaxial grains with sizes of about 20 nm (Figure 2). The films were transparent and slightly greenish.

The composition of the films was controlled by atomic absorption spectroscopy in a Perkin-Elmer spectrometer and by electron-probe X-ray microanalysis with a Tescan Vega TS5130 MM scanning electron microscope (SEM) equipped by energy dispersive X-ray spectrometer (Oxford Instruments). TEM studies were performed using JEM-4000FX microscope at an accelerating voltage of 400 kV. X-ray diffraction (XRD) was studied using a Siemens diffractometer with a graphite monochromator and a gas flow detector using Fe Kα radiation. The grain size in pure and doped ZnO was measured by TEM and additionally by XRD. It was calculated from the angular dependence of the line broadening [17]. The magnetic properties were measured using a SQUID interferometer (Quantum Design MPMS-7 and MPMS-XL) in the external magnetic field applied parallel to the sample plane. The diamagnetic signal from a sample holder and a substrate was accurately subtracted from the magnetization curves.

**Figure 3:**
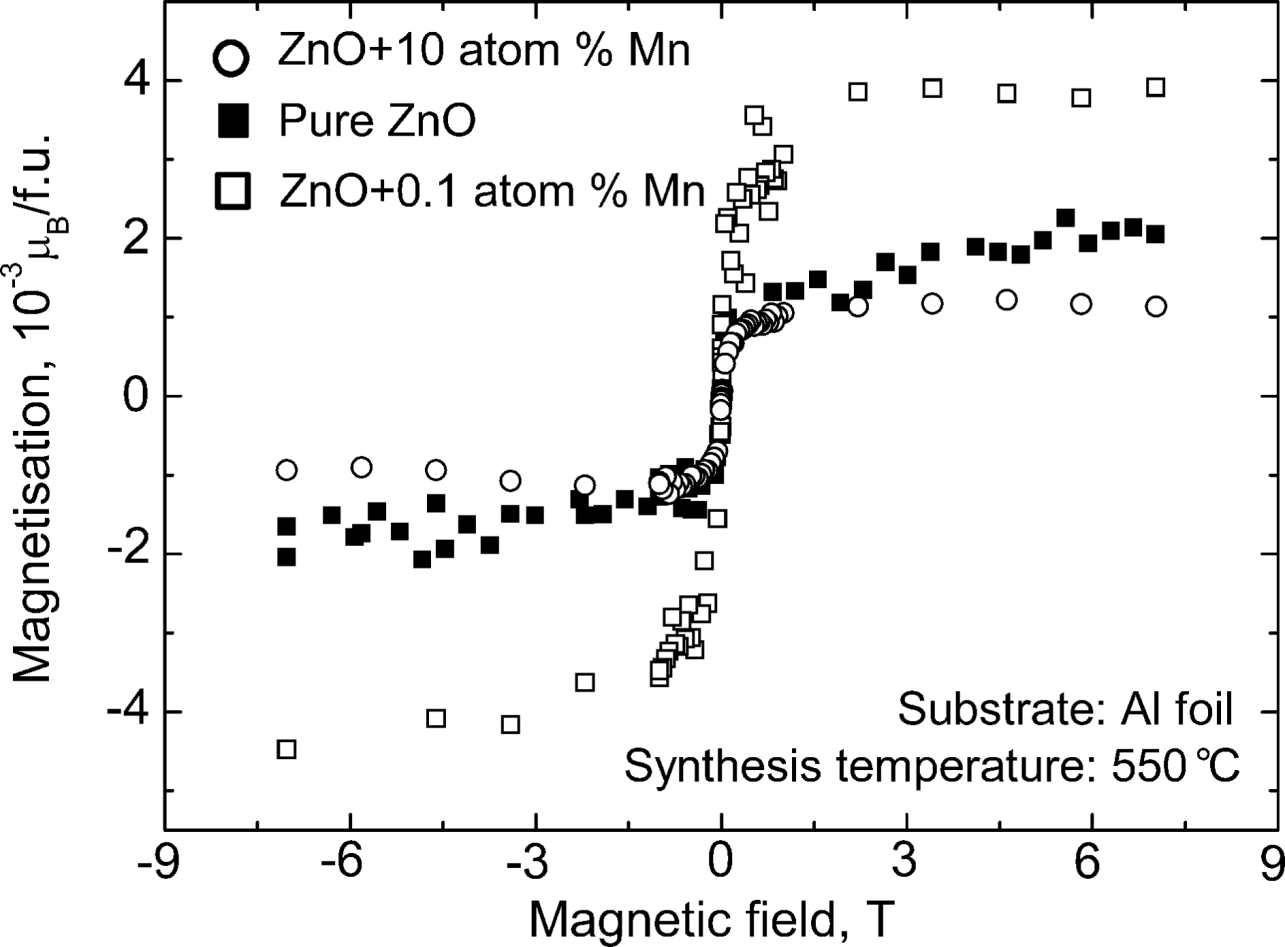
Magnetization *J*_s_ (in units of 10^−3^ µB/f.u.) as a function of the applied external magnetic field for pure zinc oxide films and zinc oxide films doped with 0.1 and 10 atom % Mn at room temperature. Reproduced with permission from [7], copyright 2009 American Physical Society.

**Figure 4:**
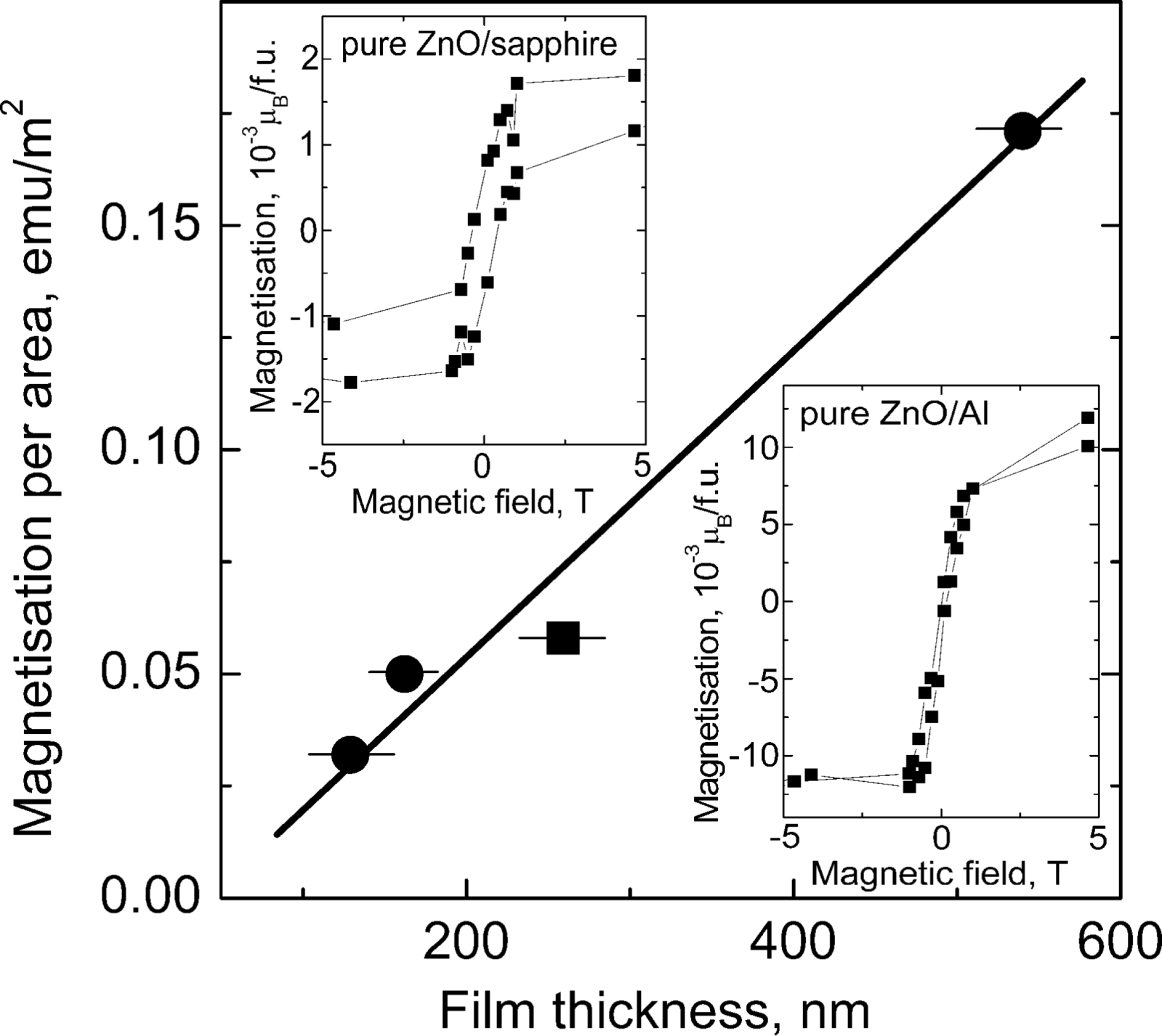
Dependence of magnetization per area unit (calibrated in emu/m^2^) on the film thickness (circles are pure zinc oxide, square is for ZnO doped by 10 atom % Mn) measured at room temperature. Insets show magnetic hysteresis for pure ZnO deposited on the sapphire single crystal (left) and on the aluminium polycrystal (right). Reproduced with permission from [7], copyright 2009 American Physical Society.

In Figure 3 the magnetization curves are plotted for pure ZnO and ZnO-doped with 0.1 and 10 atom % Mn [7]. All three curves demonstrated typical ferromagnetic behaviour with saturation (the saturation magnetization *J*_s_ is, respectively, 1 × 10^−3^ μB/f.u. = 0.06 emu/g, 2 × 10^−3^ μB/f.u. = 0.16 emu/g, and 0.8 × 10^−3^ μB/f.u. = 0.04 emu/g) and hysteresis with a coercive force *H*_c_ of about 0.01–0.02 T (see insets in Figure 4). All three samples have grain sizes well below the barrier value (Figure 2) leading to the FM behaviour. *J*_s_ increases linearly with the increasing thickness of the ZnO film (Figure 4). The temperature dependence of *J*_s_ permits to estimate the Curie temperature *T*_C_. At room temperature the saturation magnetization of pure zinc oxide films was only 40% lower than *J*_s_ measured at 40 K [7]. It means that *T*_C_ of our films is much higher than the room temperature. The main feature of all five plots in Figure 1 is that ZnO becomes ferromagnetic only if *s*_GB_ exceeds a certain critical value *s*_th_. In other words, FM properties appear if the grains are small enough. Moreover, one needs grain boundaries. If the ZnO powders are fine- or even nanograined, but not sintered (i.e., *p* << 1), they have few GBs. Then they are not ferromagnetic and, as a result, appear in the left part of a diagram.

From Figure 1 follow two important contradictions with the seminal prediction of T. Dietl [1]: (1) bulk ZnO, even doped with “magnetic” atoms, is not ferromagnetic; (2) even undoped pure ZnO can become ferromagnetic if it contains enough grain boundaries. Indeed, pure ZnO possesses ferromagnetic properties at *s*_GB_ > *s*_th_ = 5.3 × 10^7^ m^2^/m^3^ [7], in other words at grain sizes below 20 nm (Figure 1a). However, the addition of manganese, cobalt, iron and nickel positively influences the FM of ZnO polycrystals. Such additions decrease the amount of GBs needed for FM behaviour. This fact somehow coincides with the prediction of Dietl et al. [1]. For example, in a number of works where *s*_GB_ fell between *s*_th_ for pure and manganese-doped ZnO, paramagnetic properties were observed in pure zinc oxide and ferromagnetic properties in manganese-doped samples [18–19]. As a result, *s*_th_ increases with doping starting from pure ZnO. The following *s*_th_ values for different dopants have been observed: pure ZnO *s*_th_ = 5.3 × 10^7^ m^2^/m^3^ [7], cobalt-doped ZnO, *s*_th_ = 1.5 × 10^6^ m^2^/m^3^ [8], manganese-doped ZnO, *s*_th_ = 2.4 × 10^5^ m^2^/m^3^ [7], nickel-doped ZnO, is *s*_th_ = 1.0 × 10^6^ m^2^/m^3^ [6] and iron-doped ZnO, *s*_th_ = 5 × 10^4^ m^2^/m^3^ [9]. Thus, iron most actively promotes the FM behaviour of zinc oxide. ZnO polycrystals doped with Fe become ferromagnetic already at an effective grain size of about 40 μm.

### Direct evidence of grain boundary influence on the ferromagnetic behaviour of ZnO

Figure 1 shows the correlation between grain size (or specific density *s*_GB_ of grain boundaries in the volume unit) and the presence or absence of ferromagnetic behaviour in ZnO. These plots are based on the data collected from hundreds independent investigations and show that FM appears only above a certain critical value *s*_th_ of the GB specific density *s*_GB_. This is impressive evidence that GBs are the key to FM in ZnO. However, this evidence is indirect. Can we find the method that would be able to give us the direct and unambiguous evidence that ferromagnetic properties in ZnO derives from GBs?

Such direct evidence can be obtained from the local-probe method of low-energy muon spin relaxation (LE-µSR) [13]. This method is based on the idea to implant spin-polarized low-energy positive muons into ZnO. Due to their positive charge, the low-energy muons are trapped in the interstitial lattice sites. The motion of the muon spin is due to the magnetic field experienced by the muon. Therefore, low-energy muons act as highly sensitive probes of magnetic fields originating from magnetic moments in their close proximity and can provide information on the local environment of the muonin a very similar way to other magnetic resonance techniques. More details on the µSR method can be found in [20–21].

Low-energy muon spin relaxation measurements were carried out at the µE4 Low-Energy Muon (LEM) beamline at the Swiss Muon Source (SµS), Paul Scherrer Institute, Switzerland [22–23]. During these measurements the positive muons were implanted into the films. The positive muons were 100% spin polarized. The spin polarization was parallel to the sample surface. The measurements were done in zero field at different temperatures of −223, −103, and 23 °C. Different sample implantation depths were also used (10 to 75 nm). No dependence on temperature or penetration depth was observed. Therefore, the µSR spectra were obtained by averaging the data obtained at different temperatures and different sample penetration depths in order to improve the signal to noise ratio.

Three different samples were investigated with different values of *s*_GB_. One sample was single crystalline (purchased from the Mateck Company, Germany) and contained, therefore, no GBs. The second sample (coarse-grained or CG) had a grain size of 65 nm and *s*_GB_ = 2.65·10^7^ m^2^/m^3^. The third sample (fine grained or FG) had small grains with a size of 31 nm and *s*_GB_ = 5.32·10^7^ m^2^/m^3^. These *s*_GB_ values are, respectively, slightly below and above the threshold value *s*_th_ = 5.3 × 10^7^ m^2^/m^3^ for pure ZnO (Figure 1a and [7]). The magnetic measurements supported the choice of three specimens. Namely, the single crystal showed only a negligibly small saturation magnetization of 2 × 10^−4^ emu/cm^3^ [13]. The CG sample was weakly ferromagnetic with *J*_s_ = 1.25 emu/cm^3^ [13]. The FG sample with the smallest grains had the highest saturation magnetization of *J*_s_ = 8.3·emu/cm^3^. The *J*_s_ values measured at 50 K and RT were very similar (like in [7]). This fact is an important indicator for true ferromagnetism in ZnO and a high Curie temperature *T*_C_ [13].

**Figure 5:**
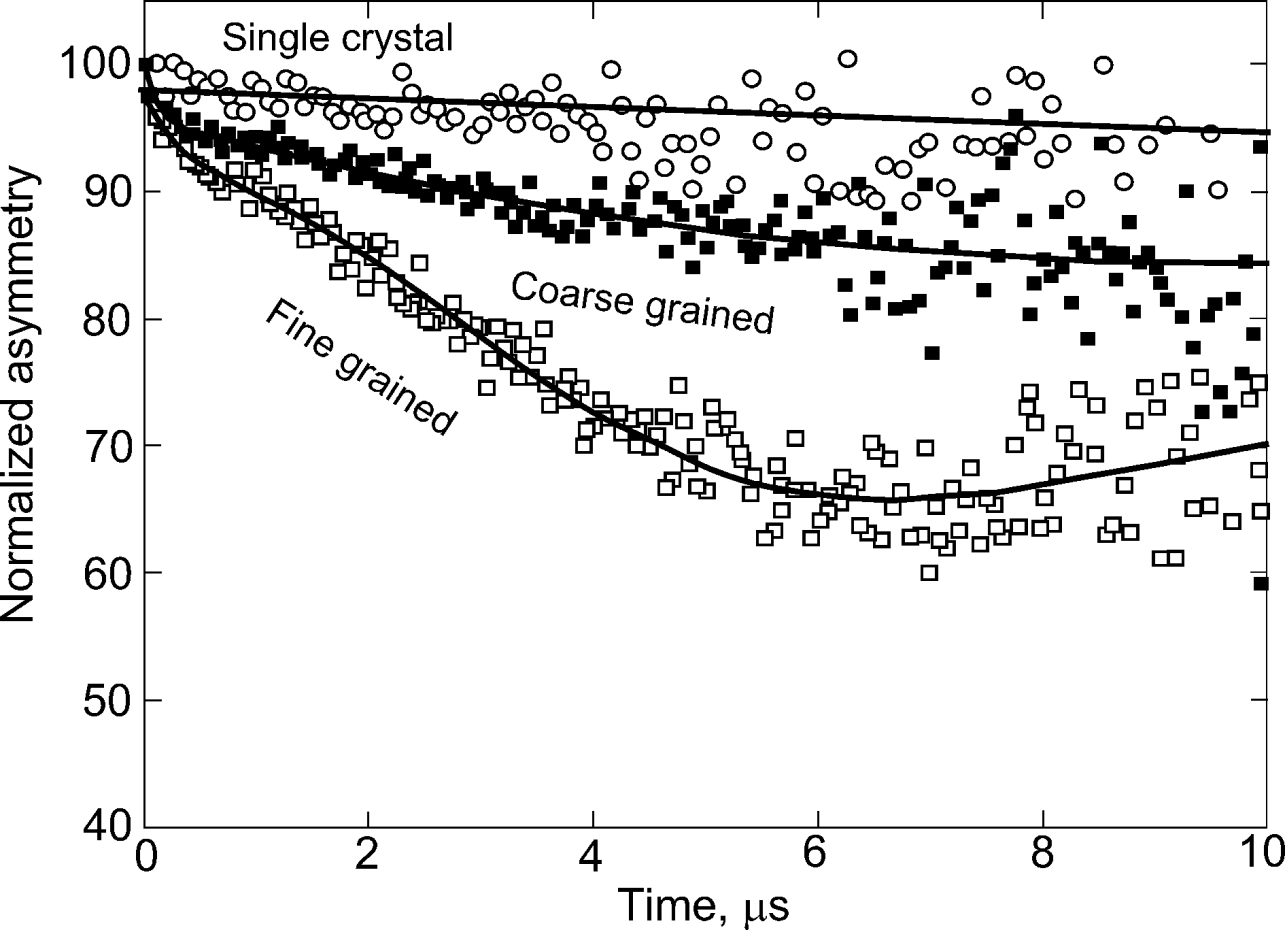
Averaged zero-field µSR spectra for the single crystal (top curve, open circles), the coarse grained (middle curve, filled squares), and the fine grained (bottom curve, open squares) ZnO samples. Replotted with permission from [13], copyright 2015 Nature Publishing Group.

When using LE-µSR the spin relaxation of muons in zero field (ZF-µSR) is measured. It shows the dephasing of muons and permits to determine the corresponding decay in the muon asymmetry spectrum [24]. As a result the decay in the muon asymmetry spectrum can be obtained. Such decay is due to the presence of an internal magnetic field distribution. In Figure 5 one can see three such time-dependent spectra of µSR asymmetry for the three studied samples with different GB specific density. In Figure 5 we plotted the normalized asymmetry. The experimental points have broad scatter and were fitted using the program Musrfit [25]. A measure for the fraction of magnetic volume in a sample is given by the relaxing amplitude of the asymmetry. The strongest relaxation is observed for the ZnO films with smallest grains (lower curve, open squares). It corresponds to a total magnetic volume fraction of about 35%. The lowest relaxation is observed for the ZnO single crystal (upper curve, open circles).The non-magnetic single crystal has no significant magnetic volume fraction at all. In the middle lies the curve for coarse-grained ZnO (filled squares). The magnetic volume fraction for this ZnO film was about 15%. Using the local-probe method of low-energy muon spin relaxation measurements we obtained the expected direct evidence that ferromagnetic behaviour of ZnO is due to the atoms located in ZnO grain boundaries and not in the bulk [13].

The LE-µSR measurements were supported by theoretical studies [13]. Using molecular dynamics the simulations of 4800 atoms in a periodic box were performed for two grain boundaries. They permitted to simulate the atomic disorder in the grain boundary region. The simulation periodic box was first equilibrated at 300 K and constant pressure of 10^5^ Pa for 0.5 ns, then heated to 2700 K and equilibrated for 1 ns. Then it was cooled to 300 K and equilibrated for 1 ns. The atomic configurations in GBs obtained by the molecular dynamics formed the basis for the further density functional theory calculations. For the cluster with about 200 atoms in an effective electrostatic field formed by the rest of the simulated system the electronic structure was determined. The calculation show that for single-crystalline ZnO the energy difference between highest occupied molecular orbital (HOMO) and lowest unoccupied molecular orbital (LUMO) is quite high and reaches about 4 eV. However, this difference for the sample containing the disordered GB area diminishes almost to zero. Moreover, energy of the lowest magnetic triplet state for GB is only 0.2 eV higher than the closed shell ground state. Both these results permit us to conclude that unpaired electrons can exist in GBs and atomic configurations may exist where such electrons are coupled ferromagnetically [13].

### Influence of dopant concentration on the ferromagnetic behaviour of ZnO

**Figure 6:**
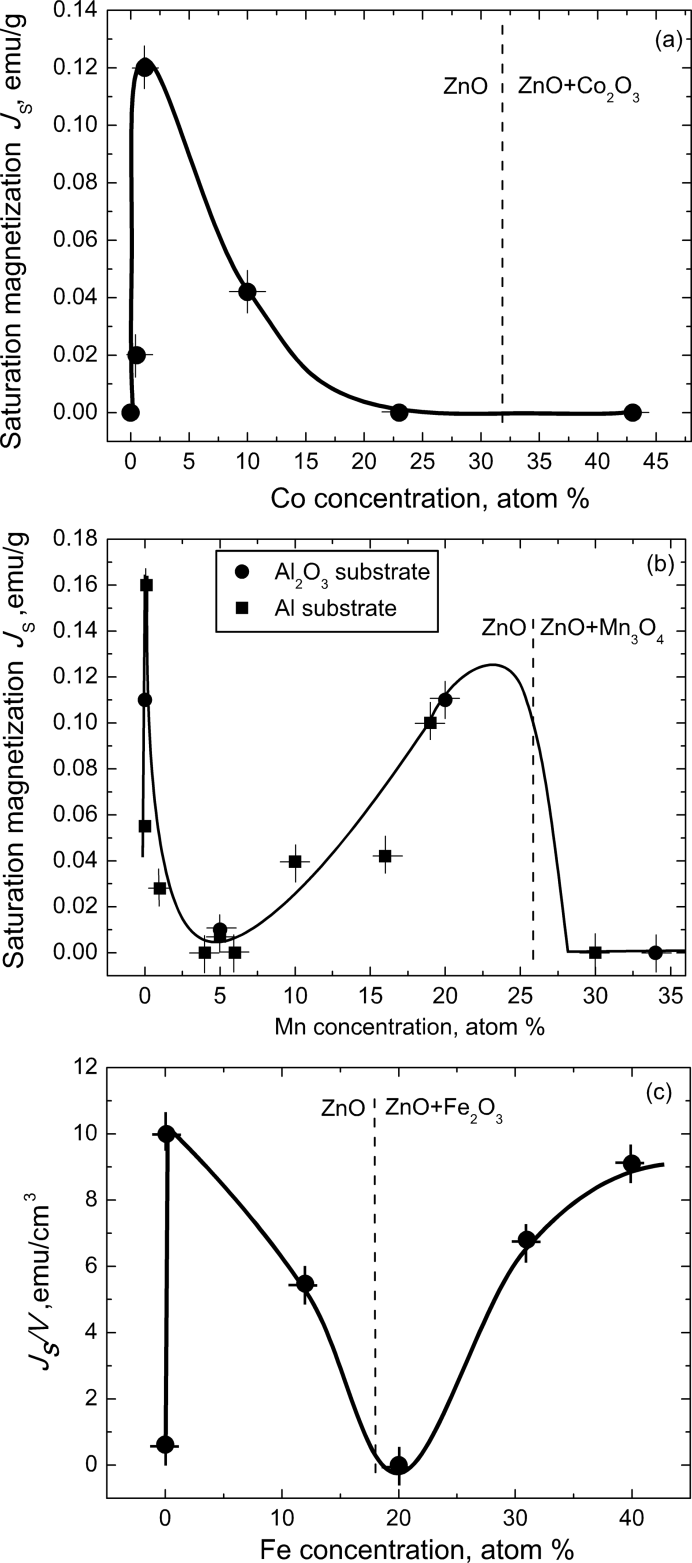
Saturation magnetization of doped zinc oxide films versus the concentration of (a) cobalt [8], (b) manganese [26], and (c) iron [9]. Figure was replotted based on the figures from [8–9,26] with permission, copyright Taylor & Francis and AIP Publishing.

In Figure 1b–e the data on presence or absence of ferromagnetic behaviour are given in dependence on the grain size for pure ZnO and ZnO doped with different atoms. However, they are given without taking in account how much manganese, cobalt, iron and nickel is in ZnO. How does the concentration of these elements influence the magnetisation of ZnO? We tried to answer this question using doped ZnO films synthesised using the liquid ceramics technology. Figure 6 shows the concentration dependences of the saturation magnetisation *J*_s_ for such ZnO films doped with manganese [26], cobalt [8], or iron [9].

In all three cases the *J*_s_ value strongly increases (about 4 to 20 times) when small fractions (0.02–0.12 atom %) of Co, Mn or Fe are added to pure ZnO. Around 0.5 atom % of Co, Mn or Fe the saturation magnetization reaches maximum and then decreases again down to the value close to that of pure ZnO or even less. Thus, the addition of small amount of “magnetic atoms” indeed makes ZnO “more ferromanetic” as predicted by Dietl et al. [1]. However, above a dopant concentration of 5–10 atom % the behaviour of *J*_s_ is different for Co, Mn and Fe. In the case of cobalt (Figure 6a), the *J*_s_(*c*) curve has only one maximum, and *J*_s_ remains low up to the solubility limit of Co in ZnO (shown by the vertical dotted line at 32 atom % Co). In the case of iron (Figure 6c), *J*_s_ increases again above solubility limit of Fe in ZnO (shown by the vertical dotted line at 18 atom % Fe) and the *J*_s_(*c*) curve has two maxima. In the case of manganese (Figure 6b), *J*_s_ strongly increases again above 5 atom % Mn, reaches a maximum close to the solubility limit of Mn in ZnO (shown by the vertical dotted line at 26 atom % Mn) and decreases for the second time down to the value for pure ZnO or less above the solubility limit of Mn in ZnO. Thus, the *J*_s_(*c*) curve has two maxima and two minima.

How we can explain the different number of maxima and minima in Figure 6? Remember that manganese can possess three different oxidation states in ZnO, namely Mn^2+^, Mn^3+^, and Mn^4+^ [27–32]. Iron can be present in ZnO in the form of Fe^2+^ and Fe^3+^ ions [33–36]. It is known that the dependence of the fraction of manganese or iron ions with various valences on the manganese or iron concentration, respectively, is complicated [27–36]. Cobalt is mainly present as Co^2+^. It looks that the more possible oxidation states has the dopant, the more complex is the shape of *J*_s_(*c*) curve. It is clear that if we substitute a Zn^2+^ ion with a Co^2+^, Fe^2+^ or Mn^2+^ ion, the amount of oxygen ions O^2–^ remains the same in the structure of ZnO. If the dopant has a higher valence than Zn^2+^, the amount of oxygen ions O^2–^ should decrease to preserve the neutral charge of doped ZnO. However, if the concentration of oxygen changes, the whole structure of the nanograined zinc oxide should change, like for example the structure and properties of titanium oxide changes by the addition of dopants with different valence [37].

We compared in [8–9,26] the shape of our concentration dependencies with those observed in other published works, i.e., in samples synthesised by other methods. In the majority of cases the concentration dependencies are also non-monotonous, but depend on the topology of the GB network. Most similar to the plots shown in Figure 6 are the *J*_s_(*c*) curves obtained from poreless films with equiaxial grains. If the grains are elongated or flattened, the shape of the *J*_s_(*c*) curves is different. Most different look the *J*_s_(*c*) curves obtained in measurements with ZnO samples built of dense polycrystalline spheres loosely sintered [8–9,26].

### Increase of dopant solubility with decreasing grain size: role of grain boundaries

**Figure 7:**
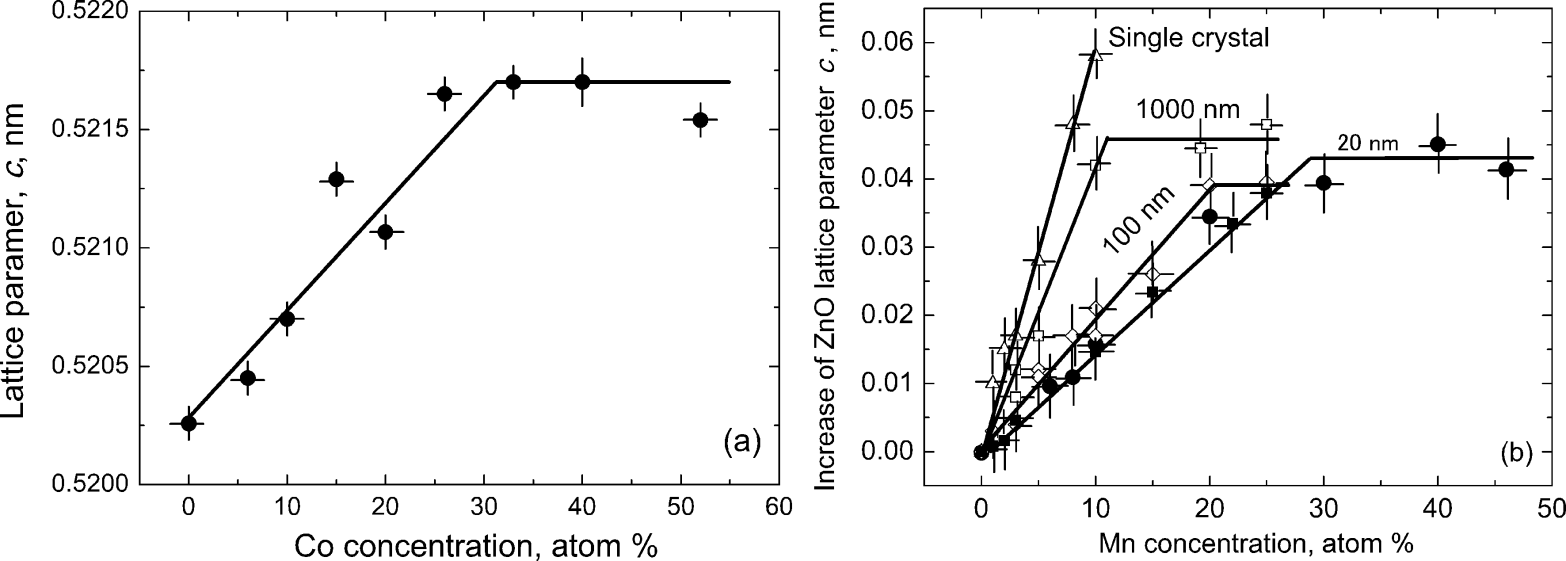
(a) Lattice parameter *c* in Co-doped ZnO films deposited using the liquid ceramics method versus the cobalt concentration [47]. (b) Period of a ZnO wurtzite lattice versus the manganese concentration for different grain size [46]. Figure was replotted basing on the figures is reproduced with permission from [46–47], copyright 2008 Elsevier Ltd. (panel a) and copyright 2009 AIP Publishing (panel b).

The vertical dotted lines in Figure 6 show the concentrations where the solubility limit *c*_s_ of Fe, Mn or Co in ZnO is reached. Above *c*_s_ a second phase appears in the system, and the peaks of Fe, Mn or Co oxide become visible in the XRD patterns together with wurtzite peaks of ZnO (Figure 7). However, why are these solubilities so high and exceed 30 atom %, for example, in the case of cobalt? Bates et al. [38] determined the temperature dependencies of the solubilities *c*_s_ of several elements (including Fe, Mn and Co) in a volume of zinc oxide. Those *c*_s_ values do not exceed few percent, even at high temperatures. In several micro- and nanograined materials the overall solubility exceeds the *c*_s_ value [39–44].

Already in 1957, McLean [45] proposed the idea that grain boundary segregation of a second component can change the overall solubility of this second component. If we add a second component B into lattice of a matrix A, the lattice parameter of A would change (like the increase of the lattice parameter of ZnO after adding cobalt atoms, Figure 7a [46]). If the bulk solubility limit *c*_sb_ is reached, a second phase will appear in addition to the first one, and the lattice parameter stops to change and remains constant with a further increase of the concentration of B. However, the atoms of the second component that are segregated in GBs cannot build the lattice of a second phase. As a result, the second phase would appear not at *c*_sb_ but later, at higher concentrations of B. This is well visible in Figure 7b where the dependence of ZnO lattice parameter is shown for different grain sizes and Mn contents [26,47]. The steepest curve is for the single crystal. The *c*_sb_ value for Mn in ZnO lattice is only about 7 atom % Mn. In this case Mn atoms only substitute Zn atoms at the wurtzite lattice sites. If we have GBs in the sample, each new Mn atom has a choice, where to substitute Zn, in the crystalline wurtzite lattice or in a GB. Thus, the curve for a grain size of 1000 nm is less steep and *c*_s_ is reached at 10 atom % Mn. With decreasing grain size and increasing specific GB area *s*_GB_ the solubility *c*_s_ limit increases further. Thus, *c*_s_ = 20 atom % Mn for a grain size of 100 nm and *c*_s_ = 28 atom % Mn for a grain size of 20 nm (Figure 7b). We see how drastically the solubility of Co and Mn increases with decreasing grain size and increasing specific GB area *s*_GB_.

The full dependencies of the lattice parameters on the dopant concentration are measured rather seldom [26,47]. However, the hundreds of papers on the ferromagnetic behaviour of ZnO give us an extremely rich source for analysing how the dopant solubility depends on the grain size. It is because when searching for ferromagnetic ZnO, the experimentalists had to be sure that the ferromagnetic signal comes from a doped wurtzite ZnO and not from a (possibly ferromagnetic) second phase. Therefore, data on the presence or absence of a second phase are usually present in such publications. Quite frequently the grain size is also given (in other case it is possible to estimate the grain size from TEM micrographs or the width of XRD peaks). The temperature *T* of synthesis or the last thermal treatment can also be determined from the publications (such as for the construction of Figure 1).

**Figure 8:**
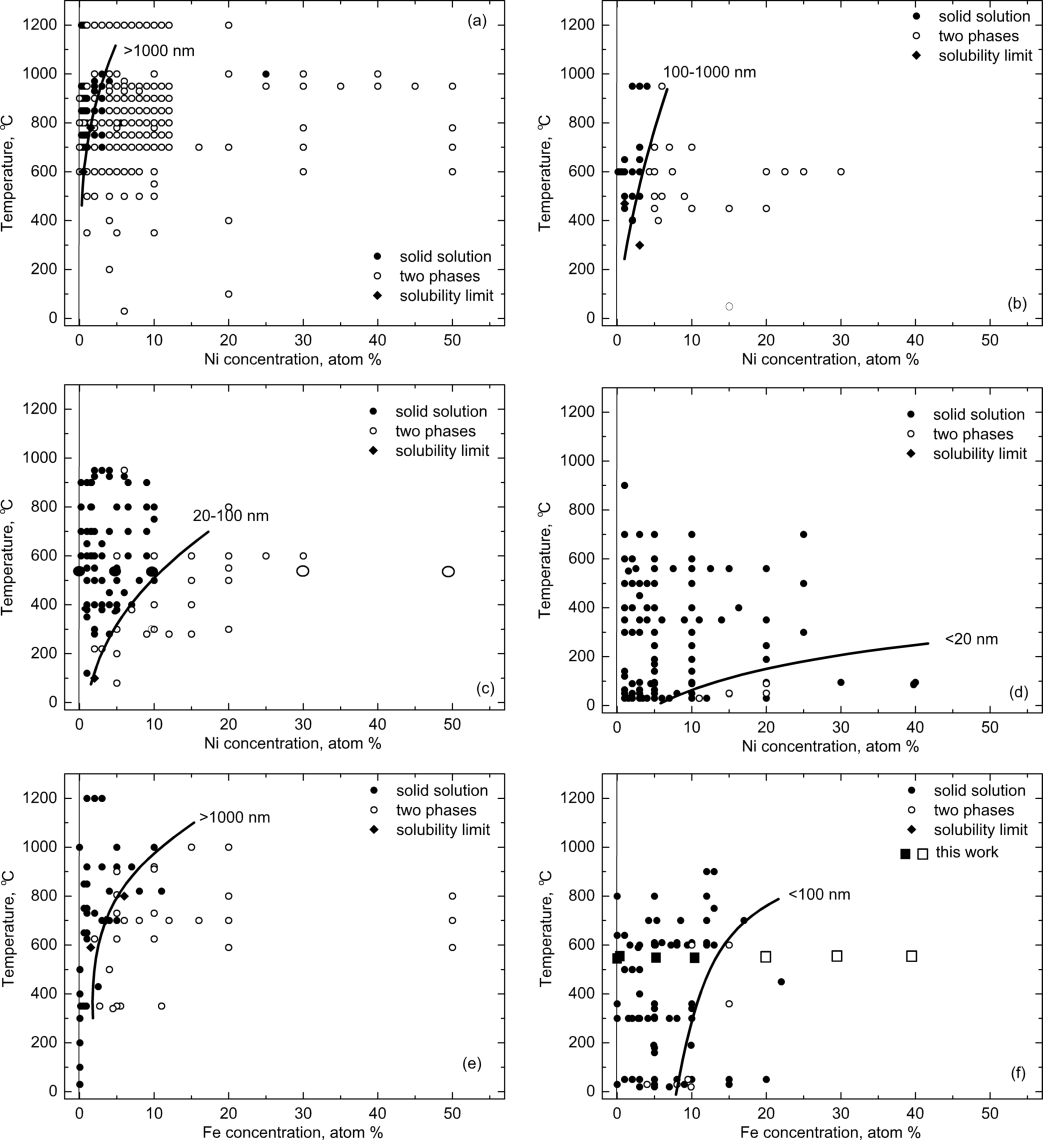
(a–d) Solubility limit of nickel in zinc oxide polycrystals with grain sizes (a) larger than 1000, (b) between 1000 and 100 nm, (c) between 20 and 100 nm, and (d) smaller than 20 nm [6]. (e,f) Solubility limit of iron in zinc oxide polycrystals with grain sizes (e) larger than 1000 and (f) smaller than 100 nm [12]. The filled and open symbols correspond to one- and two-phase samples, respectively. Diamonds mark the solubility limit. Replotted based on figures reproduced with permission from [6,12], copyright 2015 Institute of Problems of Mechanical Engineering, Russian Academy of Sciences (PME RAS, "Advanced Study Center" Co. Ltd, panels a–d) and copyright 2014 Springer Science+Business Media New York (panels e,f).

**Figure 9:**
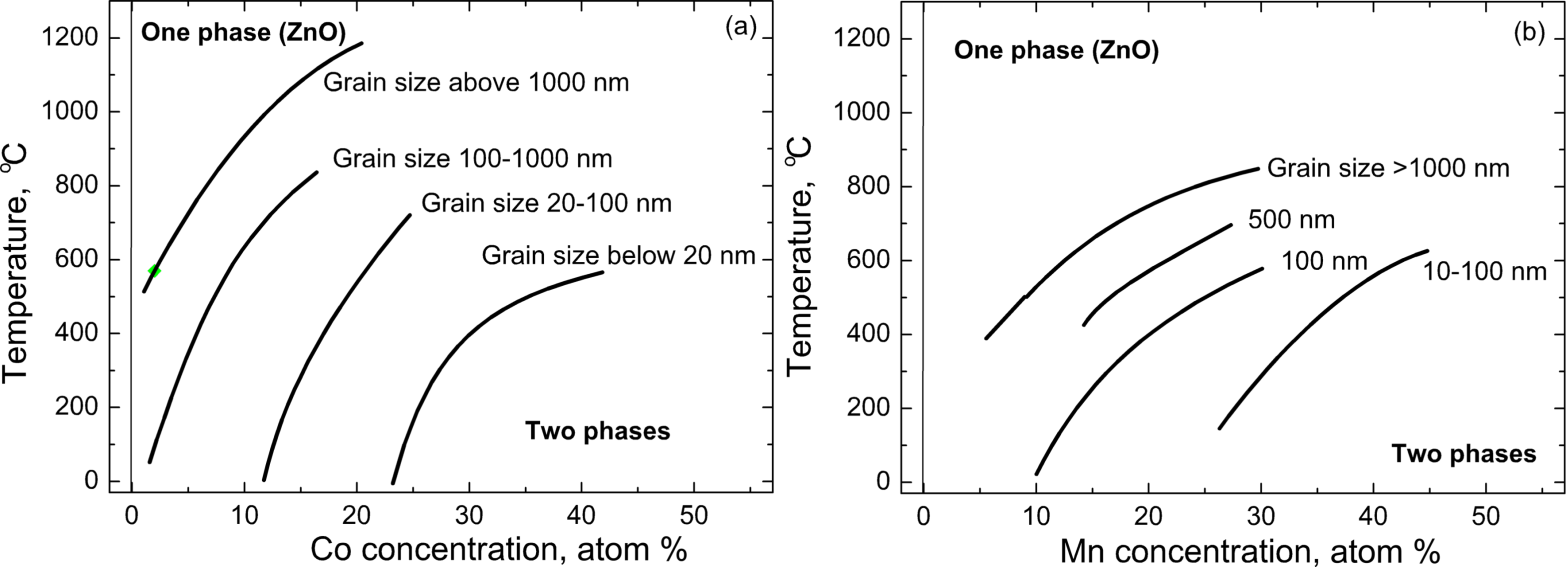
Solubility limit of (a) cobalt [47] and (b) manganese [46] in zinc oxide polycrystals with various grain sizes. Replotted based on figures reproduced with permission from [46–47], copyright 2008, 2009 Elsevier Ltd.

If we separate the data points for different grain sizes into different plots, *c*_s_(*T*) curves can be drawn for each grain size interval. Such plots for nickel-doped zinc oxide are shown in Figure 8a–d [6] and for iron-doped zinc oxide in Figure 8e,f [12]. Similar solubility lines for different values of grain size are shown in Figure 9 for manganese-doped [16] and cobalt-doped [47] ZnO polycrystals. In Figure 9 the experimental points are omitted for simplicity, and only the solubility limit lines are displayed. The full plots with all points and respective list of references can be found in [46–47]. It is well visible how drastically the solubility increases with decreasing grain size. Namely, ZnO polycrystals with grain sizes of 20 nm and below can dilute dozens of atomic per cent of “magnetic atoms” without any sign of peaks of a second phase in the XRD patterns. The loosely sintered (nano)powders contain less GBs, the main defects are free surfaces. In such samples the overall solubility also increases, but now so drastically as in poreless polycrystals [46–47].

Can the monolayer grain boundary or surface segregation ensure such a high increase of solubility? We estimated the thickness of the segregation layer for polycrystals with grain boundaries and with free surfaces [46–47]. It appeared that the GB contains more than 10 monolayers of Mn or Co [46–47]. Moreover, the GB input in the total Mn or Co concentration increases with decreasing grain size. The free surfaces are also enriched by “magnetic atoms”, but the thickness of enriched surface layers is only half to about a third of that in GBs. Can we observe these layers directly, using TEM?

**Figure 10:**
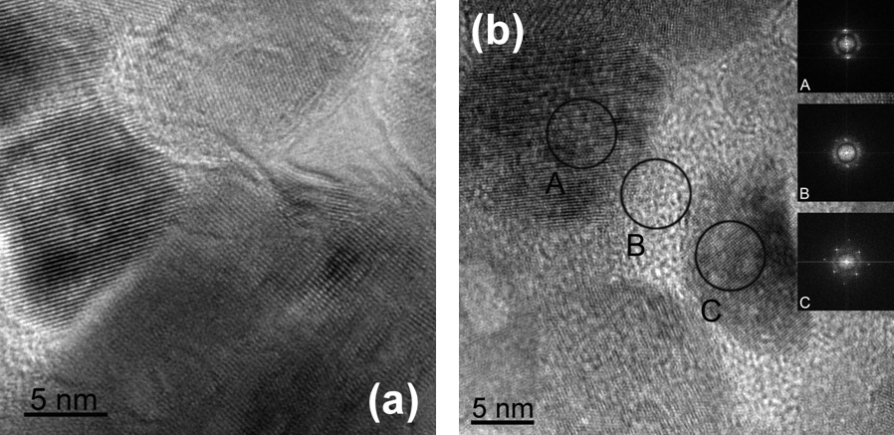
Bright-field HREM micrographs [48] for zinc oxide films doped with (a) 10 atom % Mn and (b) 15 atom % Mn. The insets show the Fourier transforms for amorphous and crystalline areas marked by letters A, B, C. Reproduced with permission from from [48], copyright 2010 Science+Business Media New York for Pleiades Publishing Inc.

Figure 10 shows the bright-field HREM micrographs for two zinc oxide films doped with 10 (Figure 10a) and 15 atom % Mn (Figure 10b) [48]. In both micrographs the ZnO nanograins are visible. They have a lattice with wurtzite structure (see the inset A with Fourier transform from crystalline area). Between crystalline ZnO nanograins the amorphous intercrystalline layers can be seen. The inset B shows the Fourier transform from such an amorphous intergranular area. It is easy to see that the amount of amorphous phase in ZnO/ZnO GBs increases with increasing manganese content. Thus, in the alloy with 10 atom % Mn the amorphous layers are visible between crystalline ZnO nanograins. In the alloy with 15 atom % Mn the crystalline ZnO nanograins are completely surrounded by amorphous layers. Such thick GB layers, indeed, correspond to the estimations made in [46–47]. However, one can find such synthesis conditions for nanograined ZnO for which, even in the case of very small grains below the threshold value (Figure 1), the sample will not have ferromagnetic properties [45–46]. The magnetic properties depend critically on the texture of films and the structure of amorphous GB layers [49–50]. Thus, the condition *s*_GB_ > *s*_th_ is necessary but not sufficient for ferromagnetism of undoped ZnO. One needs also a certain texture and structure of amorphous intercrystallite layers.

The morphology and mutual arrangement of amorphous intergranular layers and nanocrystals recalls the structures appearing in case of grain boundary wetting [51–52]. In particular, the approaches developed for the description of so-called GB complexions or intergranular films (IGFs) can be very effective in the future for the explanation and prediction of GB phenomena leading to the ferromagnetic behaviour in the nanograined semiconducting oxides [53–64]. The amorphous intergranular layers appear also in nanograined alloys obtained by the severe plastic deformation [65–66].

## Conclusion

In summary, we observed that, contrary to the prediction of Dietl et al. [1], the doping of bulk ZnO with Mn, Co, Fe or Ni does not make it ferromagnetic. On the other hand, nanograined ZnO becomes ferromagnetic even without doping. The presence of grain boundaries is the essential and necessary condition for the FM behavior of pure ZnO. The specific area of GBs *s*_GB_ has to exceed a certain critical or threshold value *s*_th_. However, the presence of grain boundaries with *s*_GB_ > *s*_th_ is not a sufficient condition for ferromagnetism of undoped ZnO. A certain texture and structure of amorphous intercrystalline layers is necessary. Nevertheless, the key role of GBs in the ferromagnetic behaviour of ZnO is proven by LE-µSR. Modelling with molecular dynamics combined with density functional theory calculations permitted to find ferromagnetically coupled electron states in ZnO GBs.

The doping of ZnO with Mn, Co, Fe or Ni, indeed, facilitates the transition into a ferromagnetic state and decreases the respective threshold values *s*_th_. Also, the addition of few tenths of atom percent of Mn, Co, Fe or Ni drastically increases the saturation magnetization *J*_s_. *J*_s_ changes non-monotonously with further increase of the dopant content *c*. The number of minima and maxima of the *J*_s_(*c*) curves correlates with number of valence states of dopants. Most probably, it is due to the change of oxygen content in GBs driven by the condition of electrical neutrality. The drastic increase of the total solubility of dopants in ZnO with decreasing grain size has been also observed. It is explained by the multilayer GB segregation.
